# Growth performance and feed utilization of keureling fish *Tor tambra* (Cyprinidae) fed formulated diet supplemented with enhanced probiotic.

**DOI:** 10.12688/f1000research.10693.1

**Published:** 2017-02-14

**Authors:** Zainal Abidin Muchlisin, Tanzil Murda, Cut Yulvizar, Irma Dewiyanti, Nur Fadli, Fardin Afrido, Mohd Nor Siti-Azizah, Abdullah A. Muhammadar

**Affiliations:** 1 Department of Aquaculture, Faculty of Marine and Fisheries, Syiah Kuala University, Banda Aceh, Indonesia; 2 Department of Biology, Faculty of Natural Science, Syiah Kuala University, Banda Aceh, Indonesia; 3 School of Biological Sciences, Universiti Sains Malaysia, Penang, Malaysia

**Keywords:** Mahseer, Probiotic, Curcuma xanthorrhiza, Kaempferia galangal, Lactobacillus casei

## Abstract

**Background**

The objective of the present study was to determine the optimum dosage of probiotic in the diet of keureling fish ( *Tor tambra*) fry.

**Methods**

*Lactobacillus casei* from Yakult® was used as a starter, and enhanced with *Curcuma xanthorrhiza*, *Kaempferia galanga* and molasses. The mixture was fermented for 7 days prior to use as probiotic in a formulated diet containing 30% crude protein. Four levels of probiotic dosage; 0 ml kg ^-1^ (control), 5 ml kg ^-1^, 10 ml kg ^-1^ and 15 ml kg ^-1^ were tested in this study. The fish was fed twice a day at 08.00 AM and 06.00 PM at the ration of 5% body weight for 80 days.

**Results**

The results showed that growth performance and feed efficiency increased with increasing probiotic dosage in the diet from control (no probiotic) to 10 ml kg ^-1^ of probiotic dosage and then decreased when the dosage was increased up to 15 ml kg ^-1^.

**Conclusions**

The best values for all measured parameters were recorded at the dosage of 10 ml kg ^-1^. Therefore, it was concluded that the optimum dosage of enhanced probiotic for *T. tambra *fry was 10 ml kg ^-1^ of feed.

## Introduction

Aquaculture is a promising business and growing faster in recent years. In Indonesia the most common species of freshwater fish used for aquaculture are several introduced species, for example, tilapia ( *Oreochromis niloticus*), common carp ( *Cyprinus carpio*), and African catfish *Clarias gariepinus* ^1, 2^. However, Indonesia has a great diversity of freshwater fish species ^3^, several of which have the potential for aquaculture. Muchlisin ^4^ evaluated 114 species of freshwater fish from the waters of Aceh Province. In total, 40 species are being utilized for consumption, and 14 species have a high economic value and great potential to be utilized as such. One of these species is *Tor tambra*, locally known as keureling fish.

Presently, aquaculture of keureling fish has already been initiated in Aceh Province, Indonesia. Several studies on this species have been documented, for example, Muchlisin ^5^ has reported the domestication techniques for broodstock, and the prevalence of ectoparasites and endoparasites in keureling fish ^6– 7^. Moreover, Muchlisin *et al.* ^8^ reported that a diet of 30% protein gave the best growth performance for *T. tambra* fry, compared to 20% and 25% protein. Muchlisin *et al.* ^9– 10^ have also studied the effect of papain enzyme and additional vitamins in the diet. However, the growth pattern analysis has shown that cultured fish display slower growth compared to wild populations ^11^. This is probably due to low protein digestibility resulting in low feed efficiency. It has been suggested that the growth performance of cultured populations could be enhanced through addition of probiotic to the diet to increase feed efficiency ^12^. Probiotic in the diet functions as an agent that triggers the metabolism of nutrients from complex compounds into simpler compounds which are readily absorbed by the intestine ^13, 14^. Several studies have reported that addition of probiotic to the diet has a significant effect on growth performance in some species of freshwater fish, for example, catfish *Pangasius sutchi* and *Pangasius hypothalamus* ^15, 16^, nile tilapia *Oreochromis niloticus* ^17^, *Catla catla* ^18^, gourami *Osphronemus gourami* ^19^ and three-spot gourami *Trichopodus trichopterus* ^20^.

It is important to overcome the low growth problem of *T. tambra* in captivity, and presently there is no study available on the effects of adding probiotic to the *T. tambra* diet. Therefore, we evaluated the effect of probiotic *Lactobacillus casei* from Yakult ^®^ enhanced with temulawak ( *Curcuma xanthorrhiza*) and kencur ( *Kaempferia galanga*) on the growth performance and feed utilization of *T. tambra.*

## Methods

### Experimental diet

The study was conducted at local aquaculture ponds at Desa Meunasah Krueng, in the Beutong Subdistrict of the Nagan Raya District, from August 2014 to December 2014. The completely randomized design was utilized in this study. Four levels of probiotic dosage were tested, namely: 0 ml probiotic kg ^-1^ of feed (control), 5 ml probiotic kg ^-1^ of feed, 10 ml probiotic kg ^-1^ of feed, and 15 ml probiotic kg ^-1^ of feed. Every treatment was replicated three times. The experimental diet containing 30% protein was prepared using raw materials purchased from the local market. Each material and formulated feed was tested for crude protein content ( Table 1).

**Table 1.** The raw materials of the formulated diet containing 30% protein used in the study.

Materials	Crude protein content of material (%)	Proportion in the diet (g kg ^-1^)	Total crude protein in the diet (%)
Fishmeal	45	210	9.45
Soybean meal	42	200	8.40
Corn flours	10	40	0.40
Fine rice-bran	9	280	2.52
Ebi shrimp meal	45	205	9.22
Cassava flours	1.5	15	0.02
Vitamins mix	-	15	-
Minerals mix	-	15	-
Soybean oil	-	20	-

### Feeding and handling of keureling fish

A total of 180 *T. tambra* fish fry with average length of 3.5 cm and average weight of 0.36 g were used in this study. The fry was purchased from the local farmer in the Nagan Raya District and distributed randomly into 12 1m × 1m × 1m hapas (cage settle nets) in a 25m × 20m × 1.3m ground pond at the stocking density of 15 fishes per hapa. The fish were weaned for 7 days prior to experimental procedures. They were fed the experimental diet minus probiotic during the weaning process. After weaning, the fish were fed on experimental diet at a feeding level of 5% body weight twice a day (08.00 AM and 06.00 PM) for 80 days. The pond was equipped with a water flow system at a water discharge of 120 L min ^-1^. The weight gain was calculated at 10 day intervals. The feces of the fish were collected from respective hapas to examine the protein content of the feces using the Kjeldahl method ^21^.

### Crude protein and lipid analysis of raw materials and feces

The crude protein in the raw material, feces and experimental diet was measured using the Kjeldahl method ^21^. About one gram of dry sample (raw material, experimental diet or feces) was weighed and placed into Kjeldahl beakers, then 10 g of catalyst (SeOCl _2_, Selenium Oxydichloride) and 25 ml sulfuric acid were added into the same beaker. The sample was heated to 250 °C for 20 minutes, shaken carefully, then heated to 350 °C for 2 hours. The samples were left to cool for 10 minutes, then 300 ml distilled water was added. Diluted samples were distilled, and this was followed by titration using 0.1 N HCl. Crude lipid was measured by chloroform-methanol extraction ^21^. Samples of the raw material or diet were homogenized with a high-speed homogenizer for 5 min and lipid was determined gravimetrically after solvent separation and vacuum drying.

### Probiotic preparation

The probiotic was prepared using a mixture of temulawak ( *C. xanthorrhiza*), kencur ( *K. galanga*), molasses and Yakult ^®^ ( *Lactobacillus casei*) as a starter. The Yakult ^®^ was purchased from a local market in Banda Aceh, Indonesia. For 1 liter of probiotic mixture the following is needed: 50 g of temulawak, 100 g kencur, 100 ml of molasses and 1 bottle of Yakult ^®^. All materials were mashed and mixed, then placed in sealed containers and fermented for 7 days. Every 2 days the container was opened to remove the gas of fermentation. For each experiment, the corresponding amount of probiotic solution was mixed with egg yolk, whisked and sprayed evenly on the diet, then dried at room temperature for 30 minutes prior to feeding to the experimental fish.

### Parameters and data analysis

Weight gain (Wg) and specific growth rate (SGR), food conversion ratio (FCR) and feed efficiency (FE) were calculated as follows:

**a.**  **Wg = Wt – Wo**, where Wg = weight gain (g), Wt = weight at the end of the experiment (g), Wo = weight at start of the experiment (g)
**b.**  The specific growth rate is the percentage of weight gain per day. **SGR (% day ^-1^) = (Ln Wt – Ln Wo) / t x 100**, where Ln= Logarithm natural, t = experiment duration (day) ^22^. Daily growth and survival rates were also calculated, based on Muchlisin *et al.* ^10^.
**c.**  The Feed Conversion Ratio is the amount of feed (g) required to produce 1 gram of fish ^23^, **FCR = F/(Wt – Wo)**, where F= the amount of given feed (g)
**d.**  The Feed Efficiency is the total weight gain produced per total weight of feed consumed ^23^, **FE (%)= (1/ FCR) x 100**.

All data were subjected to analysis of variance (ANOVA), followed by the comparison of means using Duncan’s multiple range test ^24^.

### Statement on animal ethics

All procedures involving animals were conducted in compliance with The Syiah Kuala University Research and Ethics Guidelines, Section of Animal Care and Use in Research (Ethic Code No: 958 /2015). Please refer to Supplementary File 1 for the completed ARRIVE guidelines checklist.

## Results

**Figure 1.**
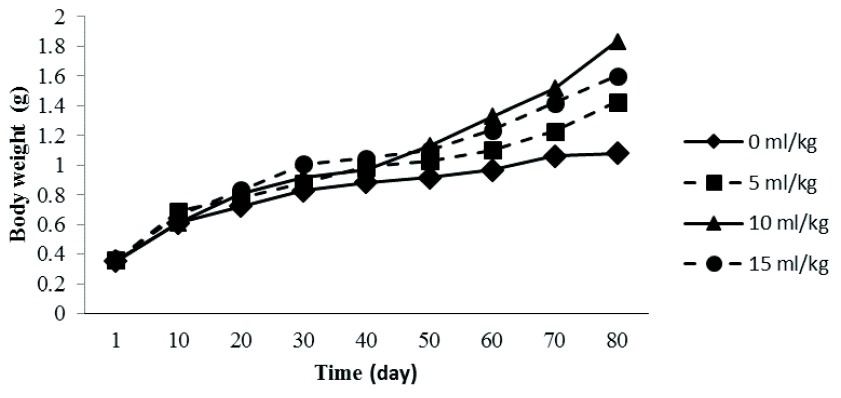
The growth trend of *T. tambra* fry at different dosage of probiotic during the study.

Estimated weight gain the keureling fish fry ranged between 0.73 g to 1.48 g, and the specific growth rate was 1.40% to 2.04%. and the survival rate was 66.67% to 95.56%( Table 2). Feed efficiency ranged between 28.40% to 42.21% and feed conversion ratio ranged between 2.37 to 3.52 ( Table 2). ANOVA revealed that probiotic had a significant effect on the growth performance, survival rate, feed efficiency, feed conversion and crude protein content in the feces of keureling fish fry (P<0.05), where the best results were observed at a dosage of 10 ml probiotic kg ^-1^ of feed. The growth trend of the keureling fish fry during the experiment significantly increased from day 10 to day 40 and from day 50 to day 80 ( Figure 1).

**Table 2.** Average (±SD) growth performance, survival rate, feed efficiency, feed conversion ratio, and protein in the feces of keureling fish fry ( *Tor tambra*) fed for 80 days at different dosage of probiotic. All data were subjected to analysis of variance (ANOVA), followed by the comparison of means using Duncan’s multiple range test. The means at different probiotic dosage across each parameter were compared. When means have the same superscript (a, b, c, d), they are not significantly different.

Probiotic dosage (ml kg ^-1^)	Weight gain (g)	Specific growth rate (% day ^-^)	Feed efficiency (%)	Feed conversion ratio	Crude protein in the feces (%)	Survival rate (%)
0	0.73 ± 0.02 ^a^	1.40 ±0.03 ^a^	28.40 ±0.27 ^a^	3.52 ± 0.33 ^c^	10.06 ± 0.03 ^c^	66.67 ±13.33 ^a^
5	1.07 ± 0.01 ^b^	1.72 ±0.02 ^b^	34.80 ±0.17 ^b^	2.87 ± 0.01 ^ab^	13.89±0.11 ^b^	68.89 ±3.85 ^a^
10	1.48 ± 0.04 ^d^	2.04±0.04 ^d^	42.21 ±0.97 ^bc^	2.37 ± 0.06 ^a^	4.53±0.11 ^a^	95.56 ±3.85 ^b^
15	1.24 ± 0.04 ^c^	1.85 ±0.06 ^c^	35.26 ±0.14 ^b^	2.84 ± 0.01 ^ab^	6.57±0.22 ^a>^	68.89 ±10.12 ^a^

## Discussion

The study revealed that a probiotic dosage of 10 ml kg ^-1^ gave the best results compared to other dosages. 10 ml kg ^-1^ may provide an optimum condition for digestive bacteria such as *Lactobacillus* sp. to grow well and facilitate feed digestibility. This is based on the low protein content in the feces, an indication that the protein was digested better at this dosage. Arief *et al.* ^25^ stated that *Lactobacillus* sp. has the ability to balance and enhance microbial condition in the digestive tract by converting carbohydrates into lactic acid, which reduces the pH and so improves the digestibility functions of the tilapia fish, *Oreochromis niloticus*. This would then stimulate the production of endogenous enzymes to improve absorption of nutrients, and inhibit the growth and activity of pathogenic organisms in the digestive tract. Irianto ^26^ also stated that the addition of probiotics to the diet increases the amount and activity of bacteria in the digestive tract of tilapia fish, and stimulates bacteria to secrete digestive enzymes such as protease and amylase which play an important role in protein and carbohydrates digestion, respectively. Marzouk *et al.* ^27^ stated that the activities of natural digestive bacteria of tilapia would change significantly when supplemented with external digestive microbes. The activity of probiotic bacteria greatly affects the balance of microflora in the digestive tract that will suppress other pathogenic bacteria resulting in increased digestive efficiency ^28^.

Temulawak and kencur contain bioactive compounds such as curcumins and atsiri oil, respectively, with associated health benefits. These compounds can function as antibiotics, neutralize toxins and increase the secretion of bile ^29^. This improves the digestive system and increases appetite in fish thus accelerating their growth performance. A similar finding by Hassan *et al.* ^30^ reported that the combination of *K. galangal* and yeast probiotic had a significant effect on the growth performance and product quality of the *Labeo rohita* fingerling. Besides, curcumin also helps promote the immune system ^31^.

However, excessive probiotics could hamper growth as recorded in this study. As observed the growth performance increased from control (without probiotic) up to 10 ml kg ^-1^ then decreased when the probiotic dosage was upped to 15 ml kg ^-1^. According to Atlas and Bartha ^32^, higher doses of probiotics favour production of secondary metabolites due to the increased bacterial load, leading to competition for nutrient and substrate utilization and inhibition of digestion and nutrient absorption. Pelczar and Chan ^33^ stated that excessive secondary metabolites will kill some bacteria groups, reducing digestibility. Therefore a number of digestive bacteria should be at an optimum level but this differs among species.

## Conclusions

Addition of probiotics to the diet of the keureling fish ( *T. tambra*) could enhance growth performance, feed efficiency, feed conversion, protein retention and protein digestibility of larva. We found that 10 ml probiotic kg ^-1^ of feed was an optimum dosage for this species.

Raw data and processed data collected for the study This includes crude protein in the feces, growth performance, daily growth rate, specific growth rate, survival rate, weight, feed conversion ratio. Click here for additional data file. Copyright: © 2017 Muchlisin ZA et al. 2017 Data associated with the article are available under the terms of the Creative Commons Zero "No rights reserved" data waiver (CC0 1.0 Public domain dedication).

Raw data of *Tor tambra* weight gain Click here for additional data file. Copyright: © 2017 Muchlisin ZA et al. 2017 Data associated with the article are available under the terms of the Creative Commons Zero "No rights reserved" data waiver (CC0 1.0 Public domain dedication).

## Data availability

The data referenced by this article are under copyright with the following copyright statement: Copyright: © 2017 Muchlisin ZA et al.

Data associated with the article are available under the terms of the Creative Commons Zero "No rights reserved" data waiver (CC0 1.0 Public domain dedication). 

**Dataset 1: Raw data and processed data collected for the study.** This includes crude protein in the feces, growth performance, daily growth rate, specific growth rate, survival rate, weight, feed conversion ratio.

DOI, 10.5256/f1000research.10693.d151536 ^34^

**Dataset 2: Raw data of *Tor tambra* weight gain.**

DOI, 10.5256/f1000research.10693.d151537 ^35^

## Supplementary material

**Supplementary Figure 1:** Completed ARRIVE guidelines checklist.
