# Supplementary material for: Growth performance and feed utilization of keureling fish Tor tambra (Cyprinidae) fed formulated diet supplemented with enhanced probiotic
Source: F1000Res. 2017 Feb 14;6:137. [Version 1] doi: 10.12688/f1000research.10693.1 (PMC5357024; doi:10.12688/f1000research.10693.1)
Supplement: Peer review history

# Peer review: Referee response for version 1

**DOI:** 10.5256/f1000research.11529.r20658

**Published:** 2017-03-13

**Authors:** Ahmad Jibril Nayaya

**Affiliations:** 1 Department of Biological Sciences, Abubakar Tafawa Balewa University (ATBU), Bauchi, Nigeria

The research was developed on the concept of probiotics being microbes that enhance good health and thereby promote optimal utilization of feed. The authors have shown that use of probiotics can improve feed uptake by the fish thereby reducing the cost of production especially where the cost of feed in some places is on the high side. 

 The title is most appropriate for the work and the Abstract gave a concise summary of the research work. Furthermore, the design and methods fit the work done. The analysis correctly explained what has been obtained from the designed work and appropriately reflects the topic studied.

 The Conclusion is balanced and has justified the basis of the results. Useful data with adequate information has been provided to warrant replication of the experiment elsewhere.

 Overall, the research has made a remarkable contribution by providing the first well documented information on positive advantage of use of probiotics in the culture of *T. tambra*.

I have read this submission. I believe that I have an appropriate level of expertise to confirm that it is of an acceptable scientific standard.

# Peer review: Referee response for version 1

**DOI:** 10.5256/f1000research.11529.r20452

**Published:** 2017-02-22

**Authors:** Usman M. Tang

**Affiliations:** 1 Faculty of Fisheries and Marine Science, Riau University, Pekanbaru, Indonesia

In my opinion, state of the art of the study has been well presented, and problem statement was also clear. We assume that authors are already familiar with this species indicate by used references. The experimental design was suitable and the data were presented very well. The results have been discussed in a good manner and compared to other previous studies.

 As known *Tor tambra* is one of the native species in Indonesia and this species has been cultured. As already mention by the authors that one the main problem in aquaculture is lack of growth rate and low feed efficiency. Therefore, application of probiotic can solve this problem, this proven by the presented data that the feed with probiotic gave higher of growth rate and feed efficiency compared to control. The new innovation of this study is using  temulawak ( *Curcuma xanthorrhiza*) and kencur ( *Kaempferia galanga*) to enhanced the probiotic. Therefore, the finding is very useful for practical use and gives a significant contribution to scientific community.

I have read this submission. I believe that I have an appropriate level of expertise to confirm that it is of an acceptable scientific standard.
